# Formulation and antimicrobial activity of a probiotic mouth freshener with phycoerythrin, *Artemisia aucheri* and encapsulated *Lactobacillus bifidus* targeting *Streptococcusmutans*

**DOI:** 10.1016/j.jobcr.2025.09.006

**Published:** 2025-09-15

**Authors:** Dorsa Salimi Manjili, Bahareh Nowruzi, Maryam Ghane

**Affiliations:** a Department of Biology, SR.C., Islamic Azad University, Tehran, Iran; b Department of Biology, IsI.C., Islamic Azad University, Islamshahr, Iran

**Keywords:** Chewable tablets, Phycoerythrin, Cyanobacteria, *Aliinostoc* sp. *1*, Encapsulated *Lactobacillus bifidus*, *Streptococcus mutans*

## Abstract

**Introduction:**

*Streptococcus mutans* is a natural flora bacterium found in the human oral cavity, responsible for tooth decay and bad breath. The rising financial costs of oral healthcare in most low- and middle-income countries have led to an increased focus on preventive measures against tooth decay using natural compounds.

**Material and method:**

The mouth freshener tablets were formulated in four treatments (T1: Tablet containing PE; T2: Tablet containing microencapsulated *L. bifidus;* T3: Tablet containing *A. aucheri* essential oil; T4: Tablet containing PE + microencapsulated *L. bifidus* + *A. aucheri* essential oil).

**Results:**

Statistical analysis of antibacterial activity showed that *S. mutans* exhibited the highest sensitivity to formulations containing + PE + *L. bifidus* + *A. aucheri.* Additionally, simulated gastrointestinal conditions over 120 min indicated the lowest and highest probiotic bacterial viability in formulations containing + PE + *L. bifidus* and +PE + *L. bifidus* + *A. aucheri*, respectively (*P* < 0.05). Histological analysis of the prepared tablets demonstrated that the addition of microencapsulated bacteria and *A*. *aucheri* essential oil significantly reduced tablet hardness, cohesion, and textural integrity (*P* < 0.05), though it did not significantly affect elasticity. Furthermore, antioxidant activity tests revealed an increase in antioxidant capacity in chewable tablets containing PE and *A*. *aucheri* essential oil. Despite the reduced sensory evaluation scores for taste and odor in PE-containing tablets, *A*. *aucheri* essential oil improved taste and odor, mitigating PE's undesirable effects. In contrast, PE significantly enhanced color and texture sensory evaluations.

**Conclusion:**

It can be concluded that chewable tablets based on PE and *A*. *aucheri* essential oil demonstrated potential to prevent tooth decay and, due to their antioxidant properties, may also have a role in managing factors related to oral ulcers. However, these findings are based on in vitro studies, and further clinical research is needed to confirm their safety in oral healthcare.

## Abbreviations list:

PEs: Phycoerythrins
CCC: Cyanobacteria culture collection
PC: Phycocyanin
APC: *Allophycocyanin*
EY: Efficacy evaluation
SEM: Scanning electron microscope
MIC: Minimum Inhibitory Concentration
MBC: Minimum Bactericidal Concentration
IRB: Institutional Review Board
SEM: Standard error of the mean
CIs: Confidence intervals
HIPEs: High internal phase emulsions

## 1 Introduction

Tooth decay is a common disease among children and adolescents in the modern era, caused by the interplay of three main factors: the host, fermentable carbohydrates, and acid-producing bacteria such as *Streptococcus mutans*. Addressing this multifactorial condition requires innovative strategies that target both the pathogenic bacteria and oxidative stress, which contribute to oral diseases.^1^

One promising natural compound is Phycoerythrins (PEs), a red pigment-protein complex derived from red algae or cyanobacteria. These pigments possess potent antioxidant, anti-inflammatory, and antimicrobial properties. Their unique protein-pigment structure enables effective scavenging of free radicals, helping counter oxidative stress implicated in oral diseases like periodontitis and dental caries. Incorporating PE into oral health formulations expands the spectrum of natural antioxidants beyond typical plant phenolics, offering a novel bioactive component rarely explored in this context.^2^

Alongside PE, *Artemisia aucheri* essential oil contains diverse bioactive compounds with well-documented antimicrobial and antioxidant activities. It can disrupt oral pathogenic microbial biofilms and reduce oxidative damage to oral tissues. Together with PE, it provides a synergistic antimicrobial effect that directly targets oral pathogens, enhancing overall efficacy.^3^ Beneficial bacteria such as encapsulated probiotics, including *Lactobacillus bifidus*, also play a crucial role in maintaining a balanced oral microbiome. These probiotics modulate microbial communities, inhibit pathogens like *Streptococcus mutans*, and support oral health through microbiome restoration. Combining PE, *A. aucheri* essential oil, and encapsulated probiotics creates a novel multi-component strategy with synergistic antimicrobial and antioxidant effects. While PE and *A. aucheri* essential oil jointly reduce oxidative stress and inhibit pathogens, encapsulated probiotics help sustain long-term oral microbiome balance.

This research is significant as it proposes an accessible and relatively low-cost adjunctive solution for preventing dental caries. However, it is important to emphasize that most currently available evidence on these compounds’ antimicrobial and antioxidant activities comes from controlled laboratory (in vitro) studies; thus, further in vivo studies and clinical trials are essential to validate efficacy and safety.

The hypothesis is that combined use of PE, *A. aucheri* essential oil, and encapsulated probiotics will yield synergistic antimicrobial and antioxidant effects, improving oral health by reducing pathogenic colonization—especially *S. mutans*—and minimizing oxidative damage. This study aims to develop and evaluate a probiotic mouth freshener containing PE extracted from the cyanobacterium *Aliinostoc* sp. 1, *A. aucheri* essential oil, and encapsulated L. bifidus, assessing its antimicrobial properties against *S. mutans*. This unique formulation provides multimodal benefits through combined antimicrobial, antioxidant, and sensory effects, offering a comprehensive oral health approach.

## 2 Methodology

### 2.1 Cultivation of *Aliinostoc* sp. *1*

The cyanobacterial species *Aliinostoc* sp. *1* was isolated from the cyanobacteria culture collection (CCC) of the ALBORZ Herbarium (sweetgum.nybg.org/science/ih/herbarium_details.php?irn=253911) at the Science and Research Branch of the Islamic Azad University in Tehran.

### 2.2 Extraction of PE

The bacterium was cultivated in modified Zarrouk medium under illumination of 300 μmol/m^2^/s at a temperature of 28 ± 2 °C for 30 days.^4^ The PE was extracted by repeated freeze-thaw cycles at −20 °C and subsequent thawing at room temperature in darkness. The extracted PE was stored at −20 to −5 °C after freeze-drying.^5^

### 2.3 Spectrophotometry analysis and quantification of PE concentration and purity

The absorption spectrum of PE was measured in the range of 250–700 nm using a UV–Vis spectrophotometer (Cary WinUV (Agilent)). The concentration of PE was calculated and reported based on the method described by Antelo et al.^6^ using the following equations:

(1) $$\text{PC}\;\left(\text{Phycocyanin}\right)\;\left(\mu g\;{\text{mL}}^{-1}\right)=\frac{\left(\text{OD}\;620\text{nm}-0.7\text{OD}\;650\text{nm}\right)}{7.38}\;)$$

(2) $$\text{APC}\;\left(Allophycocyanin\right)\;\left(\mu g\;{\text{mL}}^{-1}\right)=\frac{\left(\text{OD}\;650\text{nm}-0.19\text{OD}\;620\text{nm}\right)}{5.65}\;)$$

(3) $$\text{PE}\;\left(\mu g\;{\text{mL}}^{-1}\right)=\left(\frac{\text{OD}\;565\text{nm}-2.8\left[\text{PC}\right]-1.34\left[\text{APC}\right]}{12.7}\;\right)$$

The purity of PE was determined using spectrophotometry based on the following equation.^6^

(4) PurityofPE=(OD620)/(OD280)

OD: Optical density of PE at 620 and 280 nm.

### 2.4 Extraction of essential oil from *Artemisia aucheri*

The *A*. *aucheri* plant was collected from Sari, Iran. The obtained extract was concentrated using a water bath at 60 °C and stored in a dark glass container at 25 °C until further experimentation.^7^

### 2.5 Preparation of L. *bifidus* suspension and microencapsulation

Initially, an 18-h culture of *L. bifidus* (ATCC 29521) was prepared in 500 mL of MRS broth at 37 °C. The bacterial cells were harvested via centrifugation at 5000 rpm for 10 min at 37 °C and subsequently diluted to 100 mL using normal saline.^8^ 20 ml of the diluted microbial suspension were mixed with 100 mL of sterile 4 % sodium alginate solution (purity 95 %, Lianyungang Fengyun Seaweed Manufacturer Co).^9^

### 2.6 Efficacy evaluation of L. *bifidus* microencapsulation

Calculation of the Efficacy evaluation **(**EY) percentage was modified from that previously reported^10^ based on the following equation:

(5) $$\text{Encapsulation}\;\text{Efficiency}\;\left(\%\right)=\frac{\text{Bacteria}\;\text{in}\;\text{the}\;\text{capsule}\;\left(\text{cfu}/g\right)}{\text{Bacteria}\;\text{added}\;\text{to}\;\text{food}\;\text{suspension}\;\left(\text{cfu}/g\right)}\times 100$$

### 2.7 Particle size distribution analysis of microencapsulated L. *bifidus*

To calculate the average diameter and dispersion index, particle size distribution was measured using a Nano Zetasizer system (Malvern Instruments).^11^

### 2.8 Morphological characterization of microcapsules by microscopy

Analyses were performed using a scanning electron microscope (SEM) at various magnifications under high vacuum and voltages ranging from 5 to 20 kV.^12^

### 2.9 Preparation of probiotic mouth freshener chewable tablets

The formulation of mouth freshener tablets included gelatin and glycerol solution. Microcapsules (1 % w/w) were incorporated into the gelatin matrix along with PE (1 % w/w, purity grade 0.53) and *A*. *aucheri* essential oil (0.8 % w/w, **Pakan Bazr Company (Isfahan, Iran)**, purity 98.02 %).

**Fig. 1:**
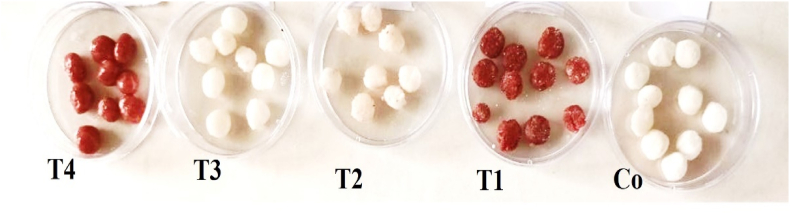
Preparation of mouth freshener tablets with different formulations according to Table 1. Co: Ctrl tablet (without *L. bifidus*, PE, and *A*. *aucheri* essential oil)/(T1: Tablet containing PE; T2: Tablet containing microencapsulated *L. bifidus;* T3: Tablet containing *A. aucheri* essential oil; T4: Tablet containing PE + microencapsulated *L. bifidus* + *A. aucheri* essential oil).

The prepared tablet bases were poured into silicone molds and refrigerated for 4 h (Fig. 1).^13^ The formulation details of the chewable tablets are presented in Table 1.

**Table 1:** Formulation of probiotic mouth freshener chewable tablets.

Treatments	Gelatin (%)	Glycerol (%)	Water (%)	PE (%)	Probiotic (%)	*A*. *aucheri* Essential Oil (%)
**Co**	7.3	8	10.3	–	–	–
**T1**	7.3	8	10.3	1	–	–
**T2**	7.3	8	10.3	–	1	–
**T3**	7.3	8	10.3	–	–	0.8
**T4**	7.3	8	10.3	1	1	0.8

Co: Ctrl tablet (without *L. bifidus*, PE, and *A*. *aucheri* essential oil)/(T1: Tablet containing PE; T2: Tablet containing microencapsulated *L. bifidus;* T3: Tablet containing *A. aucheri* essential oil; T4: Tablet containing PE + microencapsulated *L. bifidus* + *A. aucheri* essential oil).

### 2.10 Antimicrobial activity assessment of probiotic mouth freshener chewable tablets: MIC, MBC, and inhibition zone diameter

Initially, a solution of the tablets was prepared at a concentration of 6400 μg/mL and sterilized using a filter. The solution was then serially diluted in a raw ELISA plate across twelve wells containing TSB medium. Following preparation of the *S. mutans* (ATCC 35668) bacterial suspension, adjusted to a 0.5 McFarland standard, 100 μL of the bacterial suspension was added to each well, resulting in concentration ranges from 800 to 0.78 μg/mL. The plate containing bacteria was incubated at 37 °C for 24 h, after which bacterial growth was evaluated to determine MIC and MBC.^14^

For inhibition zone diameter measurement, a 5 % blood agar medium was prepared and poured into plates to a thickness of 6 mm. A bacterial suspension was prepared according to the 0.5 McFarland standard and spread across the agar surface using a sterile swab. Following incubation, the presence or absence of an inhibition zone around the disks was examined, and the inhibition zone diameter was measured in mm. Chlorhexidine was used as the positive control and water was used as the negative control in the study.^14^

### 2.11 Assessment of encapsulated *L. bifidus* viability under simulated gastric and intestinal conditions

To compare the resistance of free and alginate-encapsulated bacteria under simulated gastric and intestinal conditions, 1 g of dry capsules was mixed with distilled water and allowed to absorb moisture for 15 min. After 2 and 4 h, 1 mL of the sample was added to 9 mL of phosphate buffer to release the bacteria from microcapsules. Samples were then plated on solid MRS medium, incubated for 24–48 h, and colony counts were performed.^15^

### 2.12 Texture analysis of probiotic mouth freshener chewable tablets

Textural parameters, including firmness, springiness, and stickiness of the tablets, were measured with a texture measuring device. For this purpose, a Brookfield histometer with a cylindrical probe (Model, TA25/1000) and a speed of 2 mm/s was used. The length of the samples was 10 mm, width 10 mm and height 10 mm.^16^

### 2.13 Evaluation of antioxidant activity of probiotic mouth freshener chewable tablets

This study followed Rajauria et al.’s (2013)^17^ DPPH procedure using 96-well μl plates. The mixture contained 2.5 ml of DPPH radical solution and different concentrations of mouth freshener (100, 150, 200, 300, 500 μl). The Absorbance of ascorbic acid was used as the positive control and the absorbance of DPPH radical without any antioxidant present was used as the negative control. The reaction mixtures were kept in a dark environment at 30 °C for 30 min. Absorption measurements were conducted at a wavelength of 517 nm using a UV–Vis spectrophotometric plate reader (BioTek USA)^17^ based on the following equation:

(6) $$\%Antioxidant\;activity=\frac{\left(Control-sample\right)}{Control}\times 100$$

### 2.14 Sensory evaluation of probiotic mouth freshener chewable tablets

Thirty trained female panelists, aged 25–30 and holding a bachelor's degree, evaluated sensory characteristics using a 5-point hedonic scale (ranging from 0, indicating the most negative, to 5, indicating the most positive). The panelists were instructed to evaluate the product based on general acceptability, chewing ability, and adhesion to the wrapper, adhesion to the teeth, texture, taste, and aroma. Ethical approval was obtained from the Institutional Review Board (IRB) (Iranian National Standard No. 695, 1401).

To minimize bias in the sensory evaluation, samples were blinded and coded with randomized three digit numbers. The presentation order of the samples was randomized using a balanced Latin square design to prevent order effects and ensure that each samples was equally represented across panelists. This procedure ensured that panelists were unaware of the identity and formulation of the samples and that evaluations were based solely on sensory characteristics, thereby enhancing the objectivity and reliability of the results. Moreover, no compensation was provided for participation.^18^

### 2.15 Statistical analysis

The effect of the categorical variables on each numeric parameter were analyzed using SPSS 24, (IBM Corporation Inc.). A significance level of 95 % (*P* < 0.05) was considered to indicate statistical differences. Prior to analysis, all necessary assumptions were verified: normality was assessed using the Shapiro-Wilk test, and homogeneity of variances was evaluated by **Levene's test.** Since these assumptions were met, one way ANOVA followed by Duncan's post -hoc test was conducted to compare group's means. Each treatment was measured in triplicate and results are presented as mean ± standard error of the mean (SEM). Moreover to enhance interpretation of the results, confidence intervals (CIs) and effect sizes were reported alongside *P*-values. All experiments were performed with three replicates to ensure reliability and reproducibility of the results.

## 3 Results

### 3.1 Spectrophotometric determination of phycoerythrin concentration and purity

According to the results, the maximum average absorption of PE (1.704) was recorded at wavelengths ranging from 558 to 568 nm. Additionally, the measured concentration and purity were reported as 0.16 μg/mL and 0.53, respectively.

### 3.2 Encapsulation efficiency, particle size distribution, and morphological characterization of encapsulated *L. bifidus*

The average microencapsulation efficiency of *L. bifidus* using sodium alginate coating was reported as 97.73 ± 0.41 %.

Results obtained from the Nano Zetasizer indicated that 10 % of encapsulated *L. bifidus* had particle sizes below 1.73 μm, additionally, 50 % and 90 % of the encapsulated bacteria exhibited particle sizes below 5.32 μm and 15.18 μm, respectively. Overall, 99 % of the nanoparticles measured less than 26.46 μm (Supplementary, Fig. 1).

**Fig. 2:**
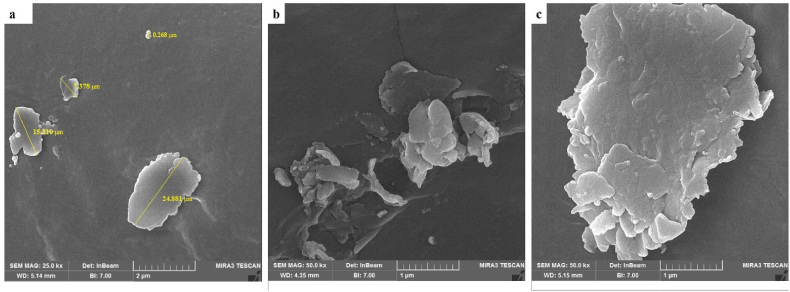
Morphology of encapsulated bacteria at magnifications of 1 μm (b, c) and 2 μm (a).

SEM microscopy analysis revealed that the microcapsules had sizes ranging from 0.268 to 24.881 μm, additionally, the microcapsule surfaces exhibited irregularities and visible cracks (Fig. 2).

### 3.3 Results of minimum inhibitory concentration (MIC), minimum bactericidal concentration (MBC), and inhibition zone diameter

Results from MIC and MBC determination, as well as inhibition zone diameter measurements of chewable tablets against *S. mutans*, revealed significant variations among treatments (*P* < 0.05). According to the findings, control tablets and tablets containing encapsulated *S. mutans* but lacking PE and *A*. *aucheri* essential oil exhibited no antibacterial activity against *S. mutans* (*P* > 0.05). Thus, MIC and MBC were identified in formulations containing + PE + *L. bifidus* + *A. aucheri* (*P* < 0.05).

Additionally, the results indicated that the smallest and largest average inhibition zone diameters of chewable tablets were observed in formulations containing + PE and +PE + LB + *A. aucheri*, respectively (Supplementary Fig. 2) (Table 2) (*P* < 0.05).

**Table 2:** Mean MIC, MBC, and inhibition zone diameter of chewable tablets against *S. mutans*.

***S. mutans***	**Co**	**T1**	**T2**	**T3**	**T4**
	MIC(mg/ml)				
	0.00 ± 0.00^d^	33.33 ± 11.74^a^	0.00 ± 0.00^d^	8.33 ± 2.94^b^	4.16 ± 1.47^c^
	MBC(mg/ml)				
	0.00 ± 0.00^c^	0.00 ± 0.00^c^	0.00 ± 0.00^c^	12.50 ± 0.00^a^	4.16 ± 1.47^b^
	Inhibition Zone Diameter (mm)				
	0.00 ± 0.00^d^	2.06 ± 0.04^c^	0.00 ± 0.00^d^	9.06 ± 0.09^b^	9.50 ± 0.07^a^

Co: Ctrl tablet (without *L. bifidus*, PE, and *A*. *aucheri* essential oil)/(T1: Tablet containing PE; T2: Tablet containing microencapsulated *L. bifidus;* T3: Tablet containing *A. aucheri* essential oil; T4: Tablet containing PE + microencapsulated *L. bifidus* + *A. aucheri* essential oil).

Different lowercase letters indicate statistically significant differences in each row (*P* < 0.05).

### 3.4 Viability of encapsulated *L. bifidus* under simulated digestive conditions

The viability results of encapsulated *L. bifidus* within chewable tablets under gastric and intestinal conditions showed varying survival rates among different treatments over time (*P* < 0.05). According to the findings, the highest viability of encapsulated *L. bifidus* in both treatments occurred within the first 10 min (*P* < 0.05). Over a 120-min period, the lowest and highest probiotic bacterial viability were observed in formulations containing + PE + *L. bifidus* and +PE + *L. bifidus* + *A. aucheri* respectively (*P* < 0.05) (Table 3) (Supplementary Fig. 3).

**Table 3:** – Mean Viability of Encapsulated L. *bifidus* (Log cfu/mL) in chewable tablets under Gastric and Intestinal Conditions.

	10 min	40 min	80 min	120 min
Gastric Conditions				
T2	9.74 ± 0.02^Aa^	9.37 ± 0.01^Ba^	7.92 ± 0.01^Cb^	6.84 ± 0.00^Db^
T4	9.74 ± 0.07^Ab^	9.10 ± 0.01^Bb^	8.29 ± 0.02^Ca^	7.39 ± 0.01^Da^
Intestinal Conditions				
T2	8.85 ± 0.001^Ab^	8.22 ± 0.05^Bb^	6.71 ± 0.011^Cb^	6.08 ± 0.002^Db^
T4	9.26 ± 0.04^Aa^	8.32 ± 0.011^Ba^	7.16 ± 0.07^Ca^	6.43 ± 0.014^Da^

T2: Tablet containing microencapsulated *L. bifidus;* T4: Tablet containing PE + microencapsulated *L. bifidus* + *A. aucheri* essential oil. Different lowercase and uppercase letters indicate statistically significant differences within rows and columns, respectively (*P* < 0.05).

### 3.5 Results of texture analysis of chewable tablets

The chewable tablet texture firmness results showed that different treatments had a significant effect on firmness (*P* < 0.05). According to the findings, the addition of PE did not significantly affect tablet firmness (*P* > 0.05). The results indicated that Ctrl and formulations containing + PE exhibited the highest firmness levels (*P* < 0.05), while the addition of microencapsulated bacteria and *A*. *aucheri* essential oil significantly reduced chewable tablet firmness (*P* < 0.05). However, no statistically significant differences were observed between formulations containing + PE + *L. bifidus;* +PE + *A. aucheri* and +PE + *L. bifidus* + *A. aucheri* (*P* > 0.05).

The chewable tablet elasticity (springiness) results indicated that different treatments did not have a significant effect on elasticity (*P* < 0.05).

The chewable tablet cohesion and consistency results showed that different treatments had a significant effect on overall cohesion and structural integrity (*P* < 0.05). According to the findings, the addition of PE, microencapsulated bacteria, and *A*. *aucheri* essential oil significantly reduced cohesion and consistency (*P* < 0.05). Ctrl Treatments exhibited the highest cohesion, whereas formulations containing + PE + *L. bifidus* + *A. aucheri* showed the lowest (*P* < 0.05) (Supplementary Table 1).

### 3.6 Results of antioxidant activity of chewable tablets

The results of the antioxidant activity of chewable tablets showed that different treatments exhibited varying antioxidant activities (*P* < 0.05). According to the findings, the Ctrl tablets and the tablets containing microencapsulated bacteria (Ctrl and +PE + *L. bifidus* treatments, respectively) demonstrated the lowest antioxidant activity (*P* < 0.05). Meanwhile, the addition of PE and *Artemisia* extract significantly increased the antioxidant activity of formulations containing + PE + *A. aucheri* and PE *+ L. bifidus* + *A. aucheri* (*P* < 0.05) (Supplementary Table 2).

### 3.7 Results of sensory evaluation of probiotic mouth freshener chewable tablets

The sensory evaluation results of chewable tablets indicated significant differences among treatments (*P* < 0.05). The addition of *A*. *aucheri* essential oil in the tablet formulation significantly enhanced the sensory evaluation of taste (*P* < 0.05). The highest taste evaluation scores were reported in formulations containing + PE + *A. aucheri* and PE *+ L. bifidus* + *A. aucheri* (*P* < 0.05), while no statistically significant differences were observed among the other treatments (*P* > 0.05).

The addition of PE significantly decreased the sensory evaluation of odor (*P* < 0.05). The lowest odor evaluation score was reported for formulations containing + PE + *L. bifidus* (*P* < 0.05). In contrast, the addition of *A*. *aucheri* essential oil significantly increased odor evaluation (*P* < 0.05), with the highest scores observed in formulations containing + PE + *A. aucheri* and PE *+ L. bifidus* + *A. aucheri* (*P* < 0.05). No statistically significant differences were reported among the other treatments (*P* > 0.05). The addition of PE significantly improved the sensory evaluation of color (*P* < 0.05), with the highest color evaluation scores reported in formulations containing + PE + *L. bifidus* and +PE + *L. bifidus* + *A. aucheri* (*P* < 0.05). No significant differences were found among the other treatments (*P* > 0.05).

The incorporation of PE, microencapsulated bacteria, and *A*. *aucheri* essential oil significantly decreased the sensory evaluation of texture (*P* < 0.05). The highest texture evaluation score was observed in the Ctrl treatments (*P* < 0.05). No significant differences were reported among the other treatments (*P* > 0.05).

The addition of *A*. *aucheri* essential oil significantly improved the overall acceptance of chewable tablets with PE *+ L. bifidus* + *A. aucheri* formulation receiving the highest scores, followed by the PE + *A. aucheri* tablets (*P* < 0.05). No statistically significant differences were observed among the other treatments (*P* > 0.05) (Supplementary Table 3).

## 4 Discussion

Phycoerythrin (PE)-gelatin complexes demonstrate favorable rheological properties that assist in stabilizing high internal phase emulsions (HIPEs) for innovative food applications. The interplay of electrostatic interactions and hydrogen bonds between PE and gelatin leads to a compact molecular structure, which improves adsorption at the oil-water interface, enhances emulsion stability, and reduces creaming in HIPEs, thereby improving extrudability.

Our findings indicate that a PE content around 3–4 % forms a dense molecular network with a solid surface membrane, resulting in enhanced mechanical strength and desirable thixotropic behavior. This is consistent with previous studies such as Matulytė et al. (2021), who reported increased firmness and reduced springiness in chewable tablets upon addition of natural compounds.^19^

Sensory evaluation showed that incorporating PE into chewable tablets reduced odor intensity, particularly when combined with L. bifidus. Conversely, the addition of Artemisia aucheri essential oil increased odor perception, likely due to its complex mixture of bioactive terpenes and related compounds.^20^ The interaction of polysaccharides and hydrophobic volatile compounds may influence aroma release and perception. It is important to note that variations in the chemical profiles of *A. aucheri* essential oil and PE can arise from differences in plant sources, extraction methods, and environmental conditions such as climate and soil composition. Such variability can significantly influence the qualitative and quantitative characteristics of bioactive extracts.^21^

Limitations of this study include potential batch-to-batch variability in natural extract composition and the need for in vivo validation of sensory and functional properties. Future research should focus on standardizing extraction methods, exploring the molecular mechanisms underlying PE-gelatin interactions, and conducting clinical trials to confirm efficacy.

## 5 Conclusion

In summary, sensory acceptance results showed that formulations containing PE, *L. bifidus*, and *A. aucheri* essential oil received the highest scores. However, a key limitation of this study remains the absence of comprehensive long-term stability data for the chewable tablets, which is critical for evaluating their commercial viability. The current stability assessments were limited primarily to tablet texture, without detailed analysis of chemical potency, dissolution profile, and moisture content. Future research should aim to conduct in-depth stability testing under various storage conditions to better understand the product's physical, chemical, and microbial durability. While the in vitro antibacterial effects of PE and A. aucheri essential oil suggest promising potential for oral healthcare applications such as toothpaste, mouthwash, and disinfectant gels, it is important to emphasize that these findings do not directly establish therapeutic efficacy. Rigorous in vivo studies and clinical trials will be necessary to substantiate these effects before any therapeutic claims can be made.

Overall, this study presents a novel multi-component formulation with synergistic antimicrobial and antioxidant properties and high sensory acceptance. Yet, the conclusions are appropriately cautious, reflecting the need for additional research to assess long-term stability, clinical efficacy, and market feasibility.

## Live vertebrates and/or higher invertebrates

This study is not on live vertebrates and/or higher invertebrates.

## Images

All the images are original and not copied from other papers.

A clear statement on informed consent obtained from all the panelists.

## Statement

We confirm that all methods were performed in **accordance** with the relevant guidelines and regulations by including a statement in the methods section.

We declared that we don't used human participants in the study.

## Ethics approval and consent to participate

Authors done experiments on mouse tissue samples. All experimental protocols and panelists involved in the study were accepted by ethics committee of Tehran medical sciences, Islamic Azad University, Tehran, Iran (IR.IAU.SRB.REC.1403.225).

## Consent for publication

Not applicable.

## Availability of data and materials

The datasets generated and/or analyzed during the current study are available in this published article.

## Authors' contributions

Conceptualization, B.N; M. GH; methodology, D. S; software, B.N.; validation, B.N.; formal analysis, B.N.

## Funding

Not applicable.

## Declaration of competing interest

The authors declare that they have no known competing financial interests or personal relationships that could have appeared to influence the work reported in this paper.

## References

[1] Jain R.L., Tandon S., Rai T.S., Mathur R., Soni K.K., Rawat M. (2022). A comparative evaluation of xylitol chewing gum and a combination of IgY+ xylitol chewable tablet on salivary streptococcus mutans count in children: a double-blind randomized controlled trial. Int J Clin Pediatr Dent.

[2] Tan H.T., Yusoff F.M., Khaw Y.S. (2022). A review on a hidden gem: phycoerythrin from blue-green algae. Mar Drugs.

[3] Hussain A., Hayat M.Q., Sahreen S., ul Ain Q., Bokhari S.A. (2017). Pharmacological promises of genus Artemisia (Asteraceae): a review: pharmacological promises of genus Artemisia. Proc Pakistan Acad Sci: B Life Environ Sci.

[4] Nowruzi B., Lorenzi A.S. (2023). Molecular phylogeny of two Aliinostoc isolates from a paddy field. Plant Systemat Evol.

[5] Mishra S.K., Shrivastav A., Pancha I., Jain D., Mishra S. (2010). Effect of preservatives for food grade C-Phycoerythrin, isolated from marine cyanobacteria Pseudanabaena sp. Int J Biol Macromol.

[6] Antelo F.S., Anschau A., Costa J.A., Kalil S.J. (2010). Extraction and purification of C-phycocyanin from Spirulina platensis in conventional and integrated aqueous two-phase systems. J Braz Chem Soc.

[7] Asl R.M.Z., Niakousari M., Gahruie H.H., Saharkhiz M.J., Khaneghah A.M. (2018). Study of two-stage ohmic hydro-extraction of essential oil from Artemisia aucheri Boiss.: antioxidant and antimicrobial characteristics. Food Res Int.

[8] Behzadnia A., Moosavi-Nasab M., Tiwari B.K. (2019). Stimulation of biosurfactant production by Lactobacillus plantarum using ultrasound. Ultrason Sonochem.

[9] Marsup P., Yeerong K., Neimkhum W. (2020). Enhancement of chemical stability and dermal delivery of Cordyceps militaris extracts by nanoemulsion. Nanomaterials.

[10] Maciel G., Chaves K., Grosso C., Gigante M. (2014). Microencapsulation of Lactobacillus acidophilus La-5 by spray-drying using sweet whey and skim milk as encapsulating materials. J Dairy Sci.

[11] Pourfarzad A., Habibi‐Najafi M.B. (2012). Optimization of a liquid improver for barbari bread: staling kinetics and relationship of texture with dough rheology and image characteristics. J Texture Stud.

[12] da Silva Júnior M.E., Araújo M.V.R.L., Martins A.C.S. (2023). Microencapsulation by spray-drying and freeze-drying of extract of phenolic compounds obtained from ciriguela peel. Sci Rep.

[13] Kazlauskaite J.A., Matulyte I., Marksa M., Bernatoniene J. (2023). Nutmeg essential oil, red clover, and liquorice extracts microencapsulation method selection for the release of active compounds from gel tablets of different bases. Pharmaceutics.

[14] Afreen S., Fatma T. (2018). Extraction, purification and characterization of phycoerythrin from Michrochaete and its biological activities. Biocatal Agric Biotechnol.

[15] Pacheco K.C., del Toro G.V., Martínez F.R., Durán-Páramo E. (2010). Viability of Lactobacillus delbrueckii under human gastrointestinal conditions simulated in vitro. Am J Agric Biol Sci.

[16] Potineni R.V., Peterson D.G. (2008). Influence of flavor solvent on flavor release and perception in sugar-free chewing gum. J Agric Food Chem.

[17] Rajauria G., Jaiswal A.K., Abu-Gannam N., Gupta S. (2013). Antimicrobial, antioxidant and free radical‐scavenging capacity of brown seaweed Himanthalia elongata from western coast of Ireland. J Food Biochem.

[18] Altuğ Onoğur T., Elmacı Y. (2011). Sensory Evaluation in Foods. İzmir, Turkey: Sidaş AOAC.(1990*)*. Official Methods of Analysis.

[19] Matulytė I. (2022).

[20] Sangian M., Soltani M., Hanifi H., Abdali N. (2022). Investigation of the effect of phycocyanin extracted from Spirulina platensis and persimmon powder on physicochemical and sensory characteristics of yogurt. Egypt J Vet Sci.

[21] Houshmand S., Alizadeh-Salteh S., Bolandnazar S., Aryakia E. (2024). Evaluating the diversity of the essential oil constituents of Artemisia accessions from Iran. J Med Plant By-Prod.

