# Supplementary material for: Formulation and antimicrobial activity of a probiotic mouth freshener with phycoerythrin, Artemisia aucheri and encapsulated Lactobacillus bifidus targeting Streptococcusmutans
Source: J Oral Biol Craniofac Res. 2025 Sep 15;15(6):1508–13. doi: 10.1016/j.jobcr.2025.09.006 (PMC12465033; doi:10.1016/j.jobcr.2025.09.006)
Supplement: Multimedia component 1 [file mmc1.docx]

**
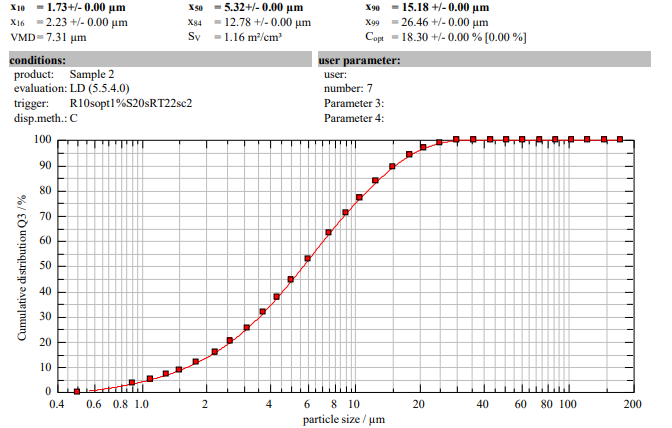
**

**Supplementary Figure 1:** Particle size distribution results of encapsulated probiotic bacteria.


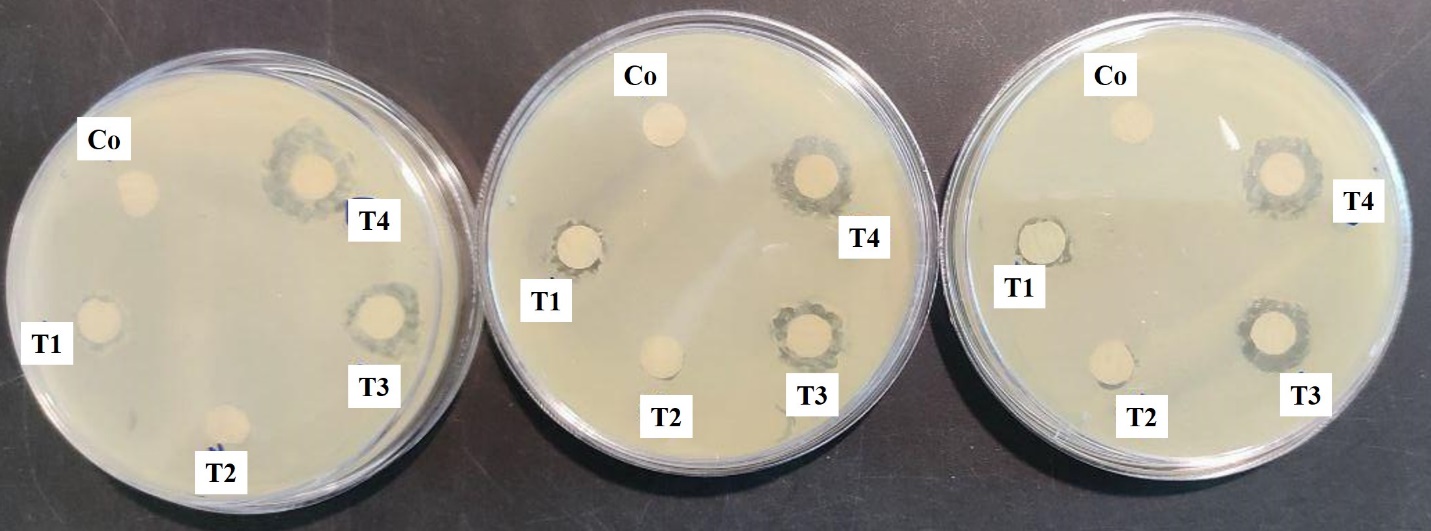


**Supplementary Figure 2:** Mean inhibition zone diameter of chewable tablets against S. mutans.

Co: Ctrl tablet (without *L.* bifidus, PE, and *A*. aucheri essential oil) / (T1: Tablet containing PE; T2: Tablet containing microencapsulated *L.* bifidus; T3: Tablet containing *A. aucheri* essential oil; T4: Tablet containing PE + microencapsulated *L. bifidus* + *A. aucheri* essential oil).

**Supplementary Figure 3:** Mean Viability of Encapsulated *L.* bifidus (Log cfu/mL) in chewable tablets under Gastric and Intestinal.

T2: Tablet containing microencapsulated *L.* bifidus; T4: Tablet containing PE + microencapsulated *L. bifidus* + *A. aucheri* essential oil).

Different lowercase letters indicate statistically significant differences in each row (p<0.05).
