# Supplementary material for: Formulation and antimicrobial activity of a probiotic mouth freshener with phycoerythrin, Artemisia aucheri and encapsulated Lactobacillus bifidus targeting Streptococcusmutans
Source: J Oral Biol Craniofac Res. 2025 Sep 15;15(6):1508–13. doi: 10.1016/j.jobcr.2025.09.006 (PMC12465033; doi:10.1016/j.jobcr.2025.09.006)
Supplement: Multimedia component 2 [file mmc2.docx]

Supplementary Table 1 – Mean Firmness (N), Springiness (mm), and Cohesion (mm) of Chewable Tablets

|  | Co | T1 | T2 | T3 | T4 |
| --- | --- | --- | --- | --- | --- |
| Firmness (N) | 8.18±0.38^a^ | 7.61±0.40^a^ | 6.72±0.31^b^ | 6.36±0. 23^b^ | 6.43±0.22^b^ |
|  | Co | T1 | T2 | T3 | T4 |
| Springiness (mm) | 1.13±0.04^a^ | 1.20±0.08^a^ | 1.30±0.14^a^ | 1.26±0.12^a^ | 1.33±0.09^a^ |
|  | Co | T1 | T2 | T3 | T4 |
| Cohesion (mm) | 0.66±0.02^a^ | 0.61±0.01^ab^ | 0.54±0.07^bc^ | 0.49±0.03^c^ | 0.39±0.02^d^ |

Co: Ctrl tablet (without *L.* bifidus, PE, and *A*. aucheri essential oil) / (T1: Tablet containing PE; T2: Tablet containing microencapsulated *L.* bifidus; T3: Tablet containing *A. aucheri* essential oil; T4: Tablet containing PE + microencapsulated *L. bifidus* + *A. aucheri* essential oil).

Different lowercase letters indicate statistically significant differences within rows (*P*<0.05).

Supplementary Table 2 – The average results of antioxidant activity (IC50=mg/ml) of chewable tablets.

|  | Co | T1 | T2 | T3 | T4 |
| --- | --- | --- | --- | --- | --- |
| Antioxidant Activity  (IC50=mg/ml) | 49.94±0. 59^a^ | 13.39±0. 13^b^ | 43.11±1. 32^a^ | 5.31±0. 03^c^ | 5.19±0. 02^c^ |

Co: Ctrl tablet (without *L.* bifidus, PE, and *A*. aucheri essential oil) / (T1: Tablet containing PE; T2: Tablet containing microencapsulated *L.* bifidus; T3: Tablet containing *A. aucheri* essential oil; T4: Tablet containing PE + microencapsulated *L. bifidus* + *A. aucheri* essential oil).

Different lowercase letters indicate statistically significant differences within rows (*P*<0.05).

Supplementary Table 3 – Mean Sensory Evaluation Scores of Chewable Tablets

| Attribute | Co | T1 | T2 | T3 | T4 |
| --- | --- | --- | --- | --- | --- |
| Taste | 2.66±0.47^b^ | 2.00±1.00^b^ | 2.66±0.47^b^ | 5.00±1.00^a^ | 5.00±1.00^a^ |
|  | **Co** | **T1** | **T2** | **T3** | **T4** |
| Odor | 3.00±1.00^b^ | 2.00±1.00^c^ | 3.33±0.47^b^ | 5.00±0.00^a^ | 5.00±0.00^a^ |
|  | **Co** | **T1** | **T2** | **T3** | **T4** |
| Color | 3.66±0.47^b^ | 5.00±0.00^a^ | 3.33±0.47^b^ | 3.66±0.47^b^ | 5.00±0.00^a^ |
|  | **Co** | **T1** | **T2** | **T3** | **T4** |
| Texture | 5.00±0.00^a^ | 3.66±0.47^b^ | 3.66±0.47^b^ | 3.33±0.47^b^ | 2.66±0.47^b^ |
|  | **Co** | **T1** | **T2** | **T3** | **T4** |
| Overall Acceptance | 3.33±0.47^b^ | 3.66±0.47^b^ | 3.33±0.47^b^ | 4.33±0.47^ab^ | 5.00±0.00^a^ |

Co: Ctrl tablet (without *L.* bifidus, PE, and *A*. aucheri essential oil) / (T1: Tablet containing PE; T2: Tablet containing microencapsulated *L.* bifidus; T3: Tablet containing *A. aucheri* essential oil; T4: Tablet containing PE + microencapsulated *L. bifidus* + *A. aucheri* essential oil).

Different lowercase letters indicate statistically significant differences within rows (*P*<0.05).
